# Double-to-Single Umbilical Artery With a Low Pulsatility Index Leading to Fetal Death: A Case Report and Review of Literature

**DOI:** 10.7759/cureus.51273

**Published:** 2023-12-29

**Authors:** Yanting Lin, Ruiling Yan, Andong He, Jie Chen, Ruiman Li

**Affiliations:** 1 Obstetrics and Gynecology, The First Affiliated Hospital of Jinan University, Guangzhou, CHN; 2 Obstetrics and Gynecology, The Affiliated Shunde Hospital of Jinan University, Guangzhou, CHN

**Keywords:** intrauterine fetal death, umbilical artery, adverse outcome, pulsatility index, single umbilical artery

## Abstract

Single umbilical artery (SUA) may be associated with adverse pregnancy outcomes, such as fetal death, emergency cesarean section, premature delivery, small-for-gestational-age infants, and admission to neonatal intensive care unit, and some SUAs are transformed from originally double umbilical arteries (UA). The pulsatility index (PI) can reflect the resistance of UA, and clinicians attach importance to high PI but easily overlook low levels of it. We reported one case of a pregnant woman who underwent double to single UA accompanied by low UA-PI and finally had intrauterine fetal death. Additionally, the literature regarding SUA and UA-PI is reviewed. This study aims to alert clinicians to the risk of double-to-single UA with low UA-PI and strengthen fetal monitoring and timely intervention. We look forward to more clinical evidence to investigate it.

## Introduction

There are two umbilical arteries and one umbilical vein in a normal umbilical cord; when there is only one umbilical artery (UA), it is called a single umbilical artery (SUA), which is the most common cord malformation with an incidence of approximately 5.3%-4.0% [1]. Its occurrence may be associated with various factors, including the proliferation, necrosis, and thrombosis of vascular smooth muscle cells, the hypercoagulable state of pregnant women, and umbilical cord torsion [2]. The SUA is prone to increased pulsation index (PI), which attracts the attention of clinicians; a low PI of SUA is easily overlooked because it is believed to mean a low blood flow resistance, although it is sometimes a sign that blood flow is close to interruption. In this study, we present one clinical case in which her fetus had double umbilical arteries in the beginning, but the umbilical arteries became SUA with low PI in the third trimester and finally had an intrauterine stillbirth.

## Case presentation

**Figure 1:**
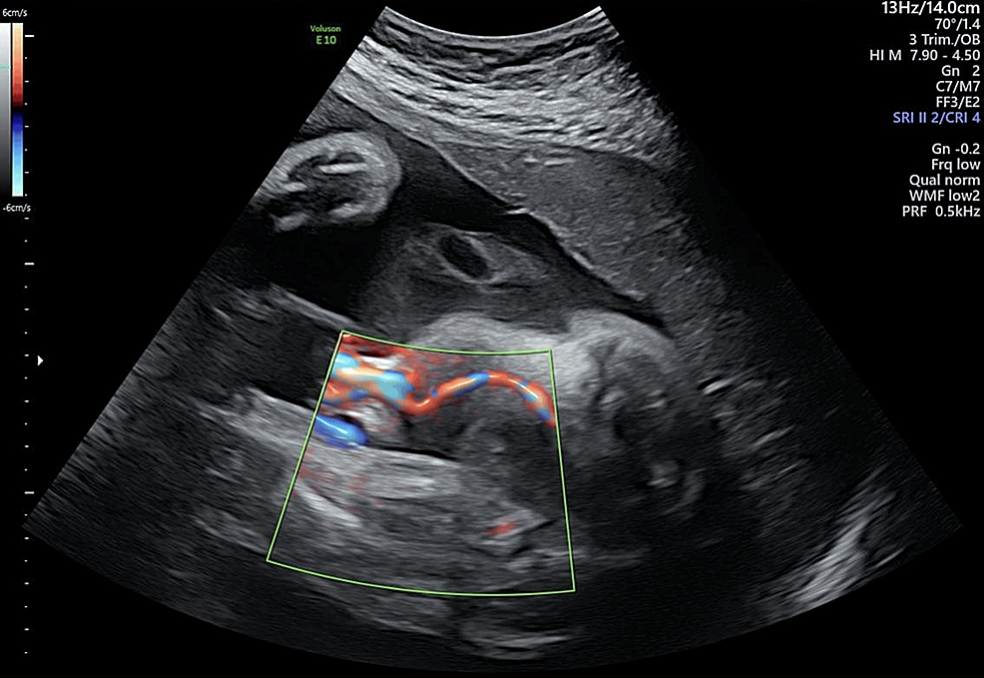
Single umbilical artery at 30+6 weeks (using a color Doppler ultrasound diagnostic system; Voluson™ E10, GE HealthCare, USA)

**Figure 2:**
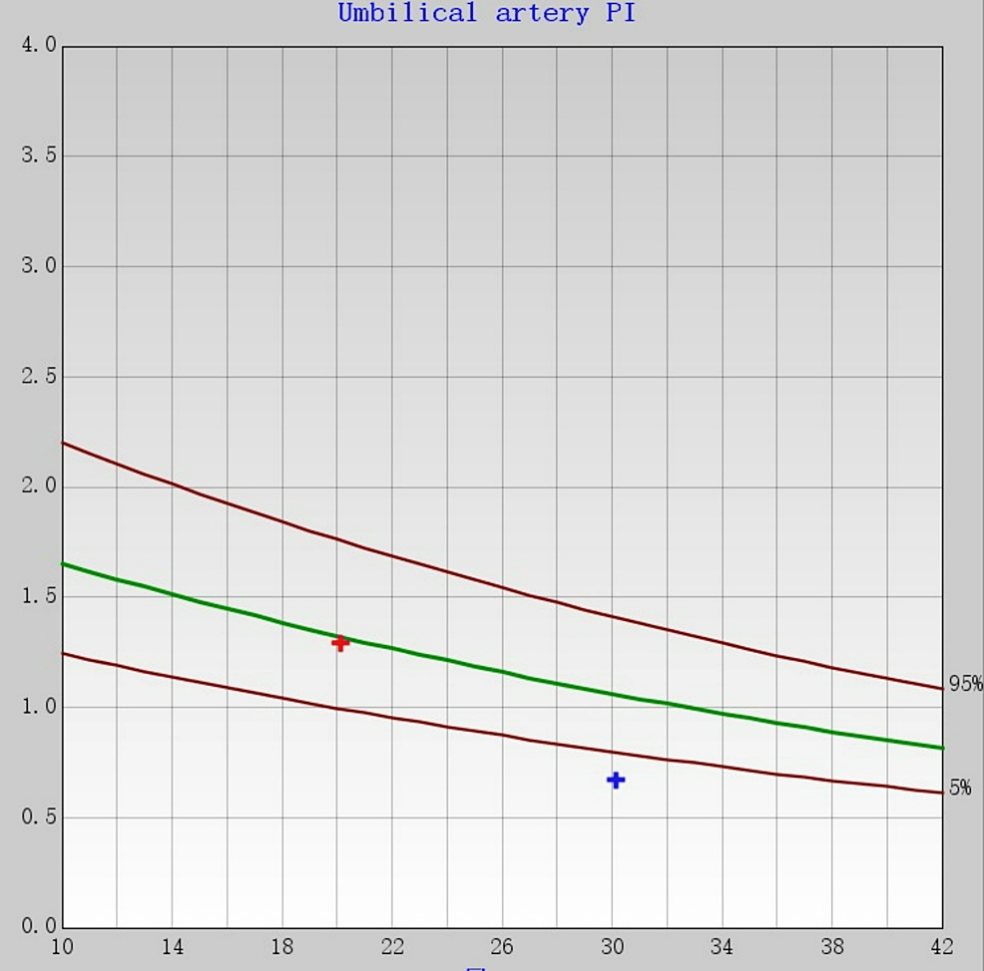
The red and blue dots indicate the corresponding umbilical artery pulsation index at that time point

In November 2021, SUA (the left side) (Figure 1) accompanied by low UA-PI at 0.67 (1.5th) and UA-resistance index (RI) at 0.48 (0.7th) was noted during regular antenatal care at 30+6 weeks of gestation in a 33-year-old multigravida (Figure 2).

**Figure 3:**
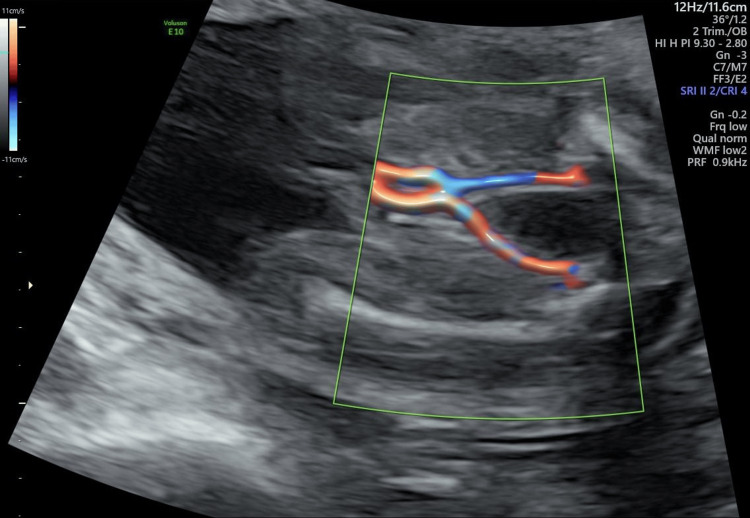
Double umbilical arteries at 20+6 weeks

Low UA-PI, i.e., UA-PI < 2th percentile for gestational age. We use a WeChat mini program Yunsuan to obtain the percentile of the UA-PI and UA-resistance index (PI) for the corresponding gestational weeks. The woman’s last pregnancy ended up with a missed abortion for an unknown reason at 13 weeks. Her progestational body mass index (BMI) was 25.32 kg/m^2^, showing she was overweight. There were no other structural abnormalities detected. Nuchal translucency was 2.9 mm at 13+3 weeks. Other routine investigations during the first and second trimesters were normal, and there were still double umbilical arteries at 20+6 weeks (Figure 3).

Due to the thick nuchal translucency, fetal two-dimensional echocardiography was done at 20+6 weeks of gestation to rule out any associated cardiac anomalies, and a four-dimensional color Doppler ultrasound was done at the same time routinely. The report was normal and no cardiovascular, urinary, or reproductive system malformations or other abnormalities were detected. The woman was operated on for amniocentesis at 31 weeks on account of the SUA found at 30+6 weeks. Because no chromosome abnormalities were found in the amniocentesis and the fear of contracting novel coronavirus, she missed her regular appointment at 33 weeks. At 34+5 weeks, she felt more than half of the fetal movement decreased, and the next day she came to our department, the hemodynamic parameters showed fetal death. The 2.10 kg stillborn female fetus’ appearance was normal. The 33 cm umbilical cord was twisted around the neck for one circle. The pathological examination showed cord edema. The patient was informed, and consent was taken before submitting this case report. Table 1 lists the details of each antenatal visit.

**Table 1:** Detailed information at each antenatal visit

Date	Gestational age (weeks)	Maternal weight (kg)	Fundal height (cm)	Maternal abdominal circumference (cm)	Fetal heart rate (bpm)	Estimated fetal weight (g)	Other findings
11/4	13+3	64.6	-	-	166	75	NT 2.9 mm
11/5	17+5	-	-	-	visible	-	The blood examinations were normal
2/6	20+6	-	-	-	157	342	Fetal echocardiography and four-dimensional Doppler ultrasound were normal
14/7	26+6	67.5	-	-	visible	-	-
4/8	29+6	68.5	27	96	150	-	-
12/8	31	69.5	28	96	145	1553	The result of the amniocentesis was normal
8/9	34+6	72	31	100	0	2293	Fetal death

## Discussion

Currently, there are two theories for the occurrence of SUA: one is that it is congenitally underdeveloped, and there is only one UA from the beginning of embryonic development [3]; the other is that there are two umbilical arteries at the beginning of embryonic development, but one of the umbilical arteries atrophies and disappears secondarily in the later development process. The potential reasons for the change of umbilical vessels from double umbilical arteries to SUA are as follows: (1) thrombosis and embolism: thrombus can form in umbilical vein or artery circulation. The etiology of umbilical vessel thrombosis can be explained by Virchow’s triad of reduced blood flow velocity, hypercoagulable state, and vascular abnormalities. The decrease in blood flow velocity may be related to mechanical damage of the umbilical cord or abnormally anatomical structure of the umbilical cord such as umbilical cord torsion, slim and narrow cord, umbilical cord entanglement, umbilical cord overlength, umbilical cord true knot, umbilical cord compression [4]. In this study, an umbilical cord twist was found in this case. Studies have shown that low UA-PI in SUA, abnormal cord blood flow, and thrombosis are mutually reinforcing, leading to a vicious cycle and ultimately vascular occlusion [5]. (2) Immune system disorder [6]: the anti-SSB/La antibody, rheumatoid factor, antinuclear and lack of protein S may be related to immune system diseases, such as primary Sjogren’s syndrome, systemic lupus erythematosus, congenital heart disease, and neonatal lupus, which are high-risk factors for thrombosis. (3) Release of inflammatory factors: inflammatory factors can lead to vascular endothelial damage, which promotes thrombosis [7]. There are other risk factors related to SUA, such as gestational hypertension, history of diabetes, smoking history, or obesity [3]. In this case, the woman's progestational body mass index (BMI) was 25.32 kg/m^2^, showing she was overweight. Due to the low incidence of double UA becoming SUA in the third trimester and the limited research data, there is no consensus on prenatal monitoring and treatment strategies. Notably, UA-PI reduction is often overlooked because the UA-PI will decrease with the increase of gestational weeks in the normal process of pregnancy, but the flow rate of UA can be extremely low when the blood flow is close to cut-off. This case resulted in intrauterine fetal death due to untimely treatment. Prenatal fetal monitoring can reduce the risk of stillbirth, including fetal movement monitoring and fetal ultrasound Doppler examination. Decreased fetal movement was found in this case. Besides, although no chromosome abnormalities were found in the amniocentesis, regular antenatal care is necessary. Therefore, this study aims to provide cautionary evidence about the clinical management of SUA, particularly SUA with low PI, to raise the profile of clinicians on it.

## Conclusions

In summary, the timely detection of UAs and their blood flow is of great importance to ensure fetal safety. Once the fetal umbilical vessels change from double UA to SUA and the UA-PI is low, clinicians may consider the possibility of UA thrombosis and embolism. However, more relevant clinical data are required to investigate the safety of low-PI SUA and its clinical management strategies.
